# Growth, Root Plasticity, and Nitrogen Allocation of *Bolboschoenus planiculmis* Under Soda Saline–Alkaline Stress

**DOI:** 10.3390/biology15151255

**Published:** 2026-07-30

**Authors:** Fengxue Shi, Hao Sun, Yingzhi Gao, Yong Wang, Chunguang He

**Affiliations:** 1 Key Laboratory of Wetland Ecology and Vegetation Restoration, Ministry of Ecology and Environment, Northeast Normal University, Changchun 130117, China; shifx816@nenu.edu.cn (F.S.);; 2 Natural History Museum of Jilin Province, Northeast Normal University, Changchun 130117, China; 3 College of Resources and Environment, Northeast Agricultural University, Harbin 150030, China; 4 National Key Laboratory of Smart Farm Technologies and Systems, Northeast Agricultural University, Harbin 150030, China; 5 Key Laboratory of Grassland Resources, Ecology of Western Arid Desert Area of the Ministry of Education, College of Grassland Science, Xinjiang Agricultural University, Urumqi 830052, China

**Keywords:** *Bolboschoenus planiculmis*, soda saline–alkaline stress, root plasticity, oxidative damage, ^15^N tracing, corm resources, waterbird habitat

## Abstract

*Bolboschoenus planiculmis* is a dominant clonal sedge in saline–alkaline wetlands of the Songnen Plain, and its corms provide important food resources for migratory waterbirds. However, its growth mechanism under saline alkali conditions is still unclear. We combined a field survey with a greenhouse experiment that decoupled saline–alkaline stress into two associated constraints, physiological drought and nitrogen deficiency. Six treatments were established: control, nitrogen deficiency, PEG-induced physiological drought, their combination, moderate saline–alkaline stress, and severe saline–alkaline stress. In the field, soil electrical conductivity and pH increased synchronously, whereas inorganic nitrogen availability was heterogeneous. In the greenhouse, compared with the CK, moderate and severe saline–alkaline stress reduced total biomass by approximately 47% and 51%, respectively, and inhibited leaf growth, root expansion, and corm biomass. It also increased SOD and CAT activities, and $H_{2}$$O_{2}$ and MDA accumulated. ^15^N tracing further showed reduced nitrogen enrichment and lower nitrogen input into corms under saline–alkaline stress, despite partial maintenance of corm allocation. Integrated trait analyses suggested that growth limitation was associated with oxidative damage, restricted morphological development, defense costs, and reduced nitrogen acquisition. These findings provide a plant-level perspective that may inform future assessments of belowground food-resource conditions in migratory waterbird stopover habitats.

## Simple Summary

The underground corms of *Bolboschoenus planiculmis* are an important food resource for migratory waterbirds in saline–alkaline wetlands of northeastern China. However, saline–alkaline habitats are stressful environments for plants because they combine salt stress, high alkalinity, water limitation, and changes in nutrient availability. In this study, we examined how *B. planiculmis* responds to saline–alkaline conditions in natural wetlands and in a greenhouse experiment. We found that saline–alkaline stress strongly reduced plant growth, restricted root development, increased physiological stress, and decreased the entry and allocation of newly absorbed nitrogen into belowground organs. Comparisons with simulated drought and nitrogen deficiency showed that saline–alkaline stress cannot be explained simply as drought or nutrient shortage alone. Instead, it acts as a complex stress involving several interacting constraints. These findings suggest that changes in belowground corm resources may be important when evaluating the ecological condition of saline–alkaline wetlands, although further field studies are needed to link plant responses directly with waterbird food availability and habitat use.

## 1. Introduction

Soil salinization and alkalization are major severe environmental challenges in arid and semi-arid regions worldwide. Soda saline–alkaline soils are particularly stressful for plants because they combine salinity-induced osmotic stress with high pH, carbonate/bicarbonate alkalinity, ion toxicity, and altered nutrient availability [1,2,3,4]. The Songnen Plain of northeastern China is one of the major soda saline–alkaline soil regions in the world, and here *Bolboschoenus planiculmis* forms extensive monodominant communities. *B. planiculmis* is a perennial clonal wetland sedge with belowground corms and rhizomes, and it is broadly distributed across temperate Eurasian wetlands, including marshes, lakeshores, riverbanks, wet meadows, and coastal or estuarine wetlands [5,6,7,8]. Its ecological significance is particularly prominent in the Songnen Plain, where its corms constitute a key energy source for the critically endangered Siberian Crane (*Leucogeranus leucogeranus*) and other migratory waterbirds during migration stopovers [7,9]. Therefore, understanding the survival mechanisms of *B. planiculmis* in saline–alkaline habitats may help link plant stress adaptation with the assessment of belowground food-resource conditions in migratory waterbird habitats.

Plant adaptation to saline–alkaline habitats is an integrated trade-off process operating across organizational levels and plant organs. A key challenge is that soda saline–alkaline stress is not a single harmful factor. Instead it includes multiple interacting constraints. High salt concentrations reduce soil water potential and restrict root water uptake, thereby inducing physiological drought [2,3,10]. Meanwhile, high pH and carbonate/bicarbonate conditions can disrupt ion homeostasis, change nutrient availability, and interfere with nutrient uptake by roots [2,3,4,11,12,13]. Nitrogen is particularly important because it is essential for plant growth, photosynthesis, antioxidant metabolism, and storage-organ formation [4,12,13]. Changes in nitrogen uptake and allocation may therefore affect balance between aboveground growth and belowground corm reserves under saline–alkaline stress [11,13]. This raises a key scientific question: in natural saline–alkaline wetlands, are the growth limitations and adaptive traits observed in *B. planiculmis* caused mainly by direct saline–alkaline toxicity, or do they also reflect responses to physiological drought and nitrogen deficiency induced by saline–alkaline conditions? Experiments based only on saline–alkaline gradients may not fully answer this question. Physiological drought and nutrient deficiency should therefore be incorporated as core secondary stress factors associated with saline–alkaline stress for mechanistic decoupling and parallel comparison.

At the physiological and resource-economics levels, plants may follow distinct resource allocation strategies in response to different types of stress. Under nutrient deficiency conditions, roots often exhibit an active foraging strategy, such as increasing specific root length or absorptive surface area to enhance nutrient capture [14,15]. In contrast, under severe osmotic stress or ion toxicity conditions, plants may shift toward a more conservative survival strategy, characterized by restricted organ expansion, altered root tissue construction, osmotic adjustment, and activation of antioxidant defense systems to mitigate cellular damage [2,3,4]. The root economics space framework provides a useful basis for interpreting such multidimensional shifts in root strategies because it links root morphology, construction cost, resource acquisition, and stress tolerance within a broader trait-based framework [16,17,18,19]. For clonal plants such as *B. planiculmis*, the adaptive strategy may be more complex because corms function as both storage and regenerative organs. Under multiple stresses, plants must balance growth, including biomass accumulation and organ development, against defense, including oxidative-stress resistance and physiological stability [20,21,22]. Recent evidence also suggests that saline–alkaline conditions can alter carbon and nitrogen allocation trade-offs in *B. planiculmis*, with consequences for clonal propagation organs and belowground resource maintenance [11]. However, it remains unclear how root morphological plasticity and nitrogen allocation are linked in *B. planiculmis* under saline–alkaline stress and its associated physiological drought and nitrogen deficiency or how these responses determine corm allocation.

Based on this background, we combined a field habitat survey in the Songnen Plain with a greenhouse decoupling experiment involving saline–alkaline stress, physiological drought, nitrogen deficiency, and combined physiological drought plus nitrogen deficiency. We aimed to address two core questions: (1) How do environmental filtering factors, particularly saline–alkaline intensity, affect phenotypic differentiation and aboveground–belowground resource allocation patterns of *B. planiculmis* in natural soda saline–alkaline wetlands? (2) How does *B. planiculmis* regulate root morphology, antioxidant defense, and cross-organ ^15^N allocation under saline–alkaline stress compared with physiological drought, nitrogen deficiency, and their combined stress?

## 2. Materials and Methods

### 2.1. Study Area and Field Sampling

#### 2.1.1. Study Area and Site Selection

**Figure 1:**
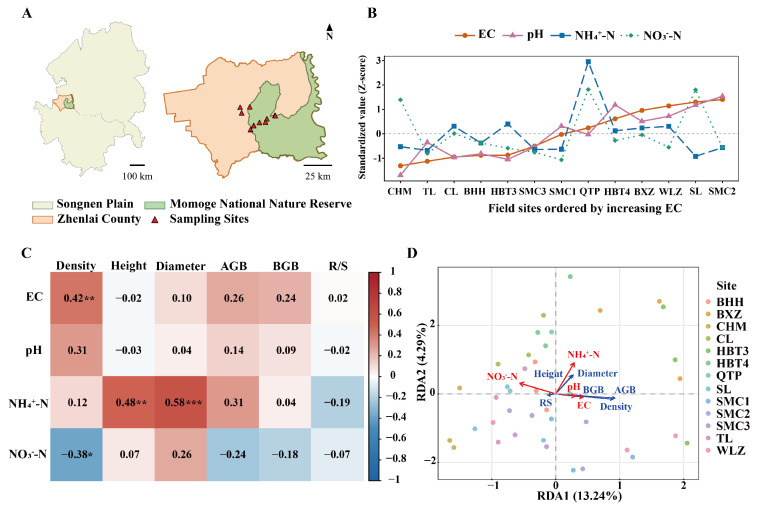
Field environmental factors in the Songnen Plain and their relationships with *B. planiculmis* phenotypes. (**A**) Geographic distribution of field sampling sites. (**B**) Standardized (Z-score) trends of core environmental factors (EC, pH, ${\mathrm{NH}}_{4}^{+}$-N, and ${\mathrm{NO}}_{3}^{-}$-N) along a gradient ordered by increasing soil electrical conductivity (EC). Solid lines represent major saline–alkaline characteristic factors, and dashed lines represent secondary nitrogen availability factors. (**C**) Spearman correlation heatmap between core environmental factors (EC; pH; ${\mathrm{NH}}_{4}^{+}$-N; ${\mathrm{NO}}_{3}^{-}$-N) and plant phenotypic traits (density, height, basal diameter, AGB, BGB, and R/S). Values in the heatmap represent correlation coefficients, and asterisks indicate statistical significance (* p<0.05, ** p<0.01, *** p<0.001). (**D**) Redundancy analysis (RDA) triplot quantitatively showing the explanatory power of compound environmental factors (red arrows) for spatial variation in plant community and individual traits (blue arrows). Points represent observations from different field sampling sites. Abbreviations: AGB, aboveground biomass; BGB, belowground biomass; R/S, root-to-shoot ratio; EC, electrical conductivity; ${\mathrm{NH}}_{4}^{+}$-N, ammonium nitrogen; ${\mathrm{NO}}_{3}^{-}$-N, nitrate nitrogen.

The field survey was conducted in the Songnen Plain of northeastern China. This region has a temperate continental monsoon climate with pronounced arid and semi-arid characteristics. The soils are dominated by carbonate-type soda saline–alkaline soils rich in NaHCO_3_ and Na_2_CO_3_, resulting in soil pH values generally ranging from 8.0 to 10.5. Based on preliminary surveys and vegetation community distribution, 13 representative natural *B. planiculmis* distribution sites were selected within natural wetlands that serve as major stopover areas for Siberian Cranes and other migratory waterbirds (Figure 1A). These sites covered a complete environmental gradient from mild to severe salinization and alkalization, defined by electrical conductivity (EC) and pH, and were intended to reflect the effects of natural environmental filtering on plant phenotypes.

#### 2.1.2. Plant and Soil Sample Collection

Field surveys and sampling were conducted during the vigorous growth period of *B. planiculmis*, from late July to early August. A gradient-based community survey method was used. At each representative sampling site, a 3 m × 3 m main plot was established in an area with uniform vegetation growth and representative community characteristics. For sample collection, three 0.5 m × 0.5 m quadrats were randomly established within each main plot.

- **Plant sampling and measurements:** Within each subquadrat, plant density was first recorded. Plant height and basal diameter were then measured in situ using a tape measure and a digital caliper. After these measurements, all aboveground plant parts in the subquadrat were clipped at the soil surface. The belowground parts, including roots and corms, were carefully excavated along the stem base, with loosely attached soil removed in the field, and then transported to the laboratory together with the aboveground samples in sealed bags on ice. In the laboratory, roots and corms were gently washed with clean water to remove adhering soil and then separated from the aboveground parts. Aboveground and belowground plant parts were separately oven-treated at 105 °C for 30 min and then dried at 65 °C to constant weight. After weighing, aboveground biomass (AGB), belowground biomass (BGB), and the root-to-shoot ratio (R/S) were calculated for each plot.
- **In situ soil sampling and processing:** To accurately match the rhizosphere microenvironment of plant growth, soil sampling was conducted simultaneously with plant excavation. At the same positions where belowground plant parts were excavated, namely, within the three subquadrats, soil samples were collected from the 0–30 cm layer using a standard soil auger. Soil samples from the three subquadrats within the same main plot were mixed in equal proportions to form one composite soil sample. After transport to the laboratory, plant residues and gravel were removed, and the soil was air-dried, ground, and sieved. Soil pH was measured by the potentiometric method at a soil:water ratio of 1:5, and soil electrical conductivity (EC) was measured using a 1:5 soil-water extract, following standard soil analytical procedures [23,24]. Soil ammonium nitrogen (${\mathrm{NH}}_{4}^{+}$-N) and nitrate nitrogen (${\mathrm{NO}}_{3}^{-}$-N) were extracted with 2 mol L^−1^ KCl and determined using a continuous-flow analyzer [25].

### 2.2. Controlled Greenhouse Experiment

#### 2.2.1. Plant Material and Pre-Cultivation

Corms of *B. planiculmis* used in this experiment were collected from typical soda saline–alkaline wetlands in the Songnen Plain. Healthy, plump corms of uniform size were selected, rinsed with deionized water, and germinated at 4 °C for 14 d. The corms were then sown in 72-cell seedling trays containing 100% vermiculite and supplied with half-strength Hoagland nutrient solution [26]. When the seedlings reached approximately 5 cm in height, healthy seedlings with uniform growth were selected and transplanted into plastic pots (26.5 cm × 19.5 cm × 20.5 cm) filled with 3 kg of a river sand and vermiculite mixture (at a 1:3 ratio). One seedling was planted in each pot. After transplanting, the seedlings were allowed to acclimate for 7 d, during which 50 mL of standard half-strength Hoagland nutrient solution was supplied twice daily, in the morning and evening. The entire controlled experiment was conducted in a greenhouse under the following conditions: temperature, 25–30 °C; relative humidity, 30–60%; photosynthetically active radiation, 400–500 μmol m^−2^ s^−1^; and photoperiod, 12 h/12 h (light/dark).

#### 2.2.2. Experimental Design and Treatments

To separate the effects of soda saline–alkaline stress on *B. planiculmis* and to distinguish the potential contributions of osmotic limitation and restricted nitrogen supply, the main stress treatment reproduced the dominant ionic composition of soda saline–alkaline soils, while physiological drought, nitrogen deficiency, and their combined treatment were included as mechanistic references. The control treatment (CK) was supplied with complete standard Hoagland nutrient solution under normal conditions (nutrient ion composition shown in Table S1). For the soda saline–alkaline treatments, a mixed salt solution was prepared according to the typical salt composition of soda saline–alkaline soils in the Songnen Plain, with a molar ratio of NaCl:Na_2_CO_3_:NaHCO_3_ = 4:2:1 [11]. Two stress levels were established: moderate saline–alkaline stress (MS), with a target salt content of 4 mg/g (salt mass/dry sand mass), and severe saline–alkaline stress (SS), with a target salt content of 8 mg/g. The physiological drought treatment (PEG) was imposed by adding 20% polyethylene glycol (PEG-6000) to the complete nutrient solution as an osmotic regulator, thereby simulating the high-osmotic-pressure environment caused by saline–alkaline stress [27]. The nitrogen-deficiency treatment (XN) used a modified nutrient solution from which all nitrogen sources were removed, ${\mathrm{NO}}_{3}^{-}$ was 0 μmol/L, while the other nutrient ions were kept the same as in the complete nutrient solution (Table S1), thereby simulating the restricted nitrogen supply that may accompany saline–alkaline conditions. The combined physiological drought and nitrogen deficiency treatment (XNPeg) simultaneously imposed 20% PEG-6000 and nitrogen-free nutrient solution to simulate a combined scenario in which osmotic limitation and restricted nitrogen supply co-occur.

The experiment was conducted as a completely randomized pot experiment with six treatments. Each treatment included 10 independent pots, with one initially uniform seedling per pot, giving a total of 60 pots. Each pot was considered one biological replicate. Within each treatment, five pots were randomly selected for ^15^N labeling, biomass determination, root architectural analysis, and ^15^N analysis, while the remaining five pots were used for physiological and biochemical measurements. To verify the actual stress conditions, substrate pH, electrical conductivity (EC), soil water content (SWC), and water potential (WP) were measured at harvest.

#### 2.2.3. Stress Application and Isotope Labeling

After the 7 d acclimation period, the first day of treatment was defined as Day 1, and the six treatment regimes were initiated simultaneously. To avoid acute osmotic shock in plants subjected to saline–alkaline stress, the mixed salt solutions for the MS and SS treatments, with concentrations of 100 g/L and 200 g/L, respectively, were applied gradually during the first three irrigations until the target salt levels were reached, with a cumulative total of 200 mL of salt solution per pot. The stress treatment lasted 30 d. During this period, the non-saline–alkaline treatments received an equal volume (200 mL) of their corresponding nutrient solutions. All pots were irrigated with water or nutrient solution every 3 d to maintain substrate moisture and stress intensity, for a total of nine irrigations.

Five pots were randomly selected from each treatment for isotope labeling and subsequent measurement. On treatment Day 10, these plants were pulse-labeled with root-applied ^15^N urea. Specifically, five injection points were established in each pot, and 0.475 g of urea solution with 10.8% ^15^N abundance, equivalent to 0.024 g of pure nitrogen, was evenly injected to a depth of approximately 5 cm below the sand surface using a long-needle syringe. This ^15^N urea labeling was used for short-term tracing of exogenous nitrogen uptake and inter-organ allocation, rather than as a long-term nitrogen supply treatment. After labeling, the original treatment conditions were maintained for another 20 d, and all labeled plants were harvested on treatment Day 30.

### 2.3. Phenotypic Traits and Physiological Measurements

#### 2.3.1. Plant Harvest and Sample Allocation

Destructive sampling was conducted on treatment Day 30. Each pot initially contained one uniform seedling; however, during the 30 d treatment period, plants produced new clonal ramets and expanded belowground corms. Therefore, all newly formed ramets and belowground organs derived from the original seedling were harvested together and treated as one pot-level biological replicate. Each treatment included 10 pots. Among them, five ^15^N-labeled pots were used for morphological measurements, biomass determination, root architectural analysis, and ^15^N analysis, while the remaining five unlabeled pots were used for physiological and biochemical measurements.

Rhizosphere sand was first gently removed, and roots and belowground organs were quickly rinsed with deionized water. Plants were then separated into three organs: leaves, roots, and corms. At this early growth stage, young rhizomes were closely connected with the fine root system and were therefore included in the root fraction for biomass determination. Thus, the root biomass reported in this study represents the combined biomass of fine roots and rhizomes, whereas corms were treated as a separate belowground storage organ. Rhizomes were not separately analyzed for morphology, physiological traits, or ^15^N allocation. For the unlabeled plants used for physiological and biochemical analyses, the separated organs were immediately frozen in liquid nitrogen and stored at −80 °C. For the labeled plants used for morphological, biomass, and ^15^N analyses, root scanning, oven-drying, weighing, and grinding were performed according to the corresponding measurement procedures.

#### 2.3.2. Aboveground Morphology, Root Architecture, and Biomass Allocation

Aboveground morphology was measured on the day of harvest. Plant height was measured from the plant base to the tip of the tallest leaf using a ruler. The number of leaves per plant was recorded, and leaf length and maximum leaf width were measured. Leaf area was estimated using a leaf-shape correction approach [28,29]. The estimated total leaf area per pot was calculated as follows: estimated total leaf area = leaf number × leaf length × leaf width × 0.75, where 0.75 was the leaf-shape correction coefficient. To characterize relative changes in leaf area deployment in different treatments, the leaf area ratio (LAR) was calculated as follows: LAR = estimated total leaf area/total plant dry biomass. Root architecture was measured at the pot level using fine roots only. After washed, fine roots were kept moist during scanning. Root images were acquired using a root scanner, and total root length, root surface area, root volume, and mean root diameter were quantified using WinRHIZO 2019 image analysis software (Regent Instruments Inc., Quebec, QC, Canada) [30]. After scanning, fine root samples, together with leaf, rhizome, and corm samples, were oven-dried for biomass determination and subsequent ^15^N analysis.

For biomass determination, leaves, fine roots, rhizomes, and corms were separately oven-treated at 105 °C for 30 min then dried at 65 °C to constant weight, after which the dry weight of each organ was recorded. Aboveground biomass (AGB) was represented by leaf biomass, while belowground biomass (BGB) was calculated as the sum of fine root, rhizome, and corm dry weights. Total biomass was calculated as AGB + BGB. The root-to-shoot ratio was calculated as follows: R/S = BGB/AGB. Root functional traits were calculated based on fine roots only: specific root length (SRL) = total root length/fine root dry weight; specific root area (SRA) = root surface area/fine root dry weight; and root tissue density (RTD) = fine root dry weight/root volume [31,32]. Biomass allocation proportions were calculated as the percentage of each organ relative to total biomass. In the biomass allocation analysis, root–rhizome biomass allocation represented the combined dry weight of fine roots and rhizomes relative to total biomass, whereas corm biomass allocation was calculated separately. Based on the morphological and biomass data of each treatment, the phenotypic plasticity index (PPI) was calculated as PPI = (maximum value − minimum value)/maximum value and was used to compare the relative plasticity of different traits along the treatment gradient [33].

#### 2.3.3. Quantification of Antioxidant Defense and Osmotic Adjustment Compounds

Physiological and biochemical indices were measured using fresh samples stored at −80 °C. Five independent biological replicates were initially assigned to each treatment. Because a small number of measurements could not be reliably quantified owing to assay failure or clear technical anomalies, the number of valid replicates varied among treatment–organ–indicator combinations. These measurements were treated as missing, and no missing values were imputed. Leaves, roots, and corms were analyzed separately. For each assay, approximately 0.1 g of fresh tissue was ground in liquid nitrogen and homogenized with 1.0 mL of the corresponding ice-cold extraction buffer supplied with the assay kit. The homogenate was centrifuged at 12,000× *g* for 10 min at 4 °C, and the supernatant was collected for colorimetric determination. Commercial biochemical assay kits (Suzhou Grace Biotechnology Co., Ltd., Suzhou, Jiangsu, China) were used to extract and determine superoxide dismutase activity, catalase activity, malondialdehyde content, hydrogen peroxide content, free proline content, soluble sugar content, and soluble protein content. These assays were based on standard colorimetric methods, including nitroblue tetrazolium reduction for SOD [34], $H_{2}$$O_{2}$ decomposition for CAT [35], thiobarbituric acid reaction for MDA [36], colorimetric determination of $H_{2}$$O_{2}$ [37], acid–ninhydrin reaction for free proline [38], anthrone colorimetry for soluble sugars [39], and Coomassie Brilliant Blue G-250 binding for soluble protein [40]. All procedures followed the standard protocols supplied with the kits, and colorimetric quantification was performed using a microplate reader. SOD and CAT activities were normalized to the soluble protein content of the corresponding samples and expressed as U mg^−1^ protein.

#### 2.3.4. Measurement and Calculation of ^15^N Stable Isotope Abundance

After harvest, ^15^N-labeled plants were separated into leaves, roots, and corms, oven-dried, and ground using a ball mill. The ^15^N atom percentage abundance (atom% ^15^N) and nitrogen mass percentage (N weight percentage, N%) of powdered samples from each organ were determined using an isotope ratio mass spectrometer (IRMS, Delta V Advantage, Thermo Fisher Scientific, Waltham, MA, USA). The atom percent excess of ^15^N (APE) in each organ was calculated as follows: APE_*i*_ = atom% ^15^N_*i*_ − atom% ^15^N_natural_, where atom% ^15^N_*i*_ represents the measured ^15^N atom percentage abundance of organ *i*, and atom% ^15^N_natural_ represents the natural abundance of ^15^N, which was set at 0.366% in this study. Here, *i* denotes leaves, roots, or corms. The amount of ^15^N absorbed by each organ was then calculated by combining organ dry weight (W_*i*_), total nitrogen content (N_*i*_%), and APE_*i*_ as follows: ^15^N_*i*_ = W_*i*_ × N_*i*_ × APE_*i*_. Finally, the allocation proportion of ^15^N among organs (F_*i*_, %) was calculated as follows: F_*i*_ = (^15^N_*i*_/∑^15^N_total_) × 100% [41,42]. The dilution or turnover of ^15^N urea in the substrate during the subsequent 20 d was not directly quantified. Therefore, the ^15^N data represent net ^15^N enrichment and inter-organ allocation in plant tissues at harvest, rather than a complete nitrogen turnover budget.

### 2.4. Statistical Analysis

Most statistical analyses and graphical visualizations were performed in R software (Version 4.5.2) [43]. The two-way ANOVA and its graphical visualization were conducted in Python using the statsmodels and Matplotlib packages. Each pot was treated as an independent biological replicate. Unless otherwise indicated, data are presented as means ± standard deviation (SD). The significance level was set at α=0.05. Prior to statistical comparisons among multiple groups, the homogeneity of variances was assessed using Levene’s test [44]. For data that met the assumption of homoscedasticity (p>0.05), one-way analysis of variance (ANOVA) followed by Tukey’s HSD post hoc test was performed to evaluate significant differences among treatments [45]. For data exhibiting heteroscedasticity (p<0.05), Welch’s ANOVA followed by the Games–Howell post hoc test was used [46,47].

For the field survey data, Spearman correlation analysis was used to assess associations between environmental factors and plant phenotypic traits across field sites [48]. To further evaluate the combined explanatory relationships between environmental factors and phenotypic variation, redundancy analysis (RDA) and principal component analysis (PCA) were performed using the vegan package [49].

Two-way ANOVA was conducted using the CK, PEG, XN, and XNPeg treatments in a 2×2 factorial design, with physiological drought (D: control versus PEG) and nitrogen supply (N: control N versus N deficiency) treated as fixed factors. Because the number of valid observations differed among treatment combinations, the D × N interaction was evaluated using a full factorial linear model with Type III sums of squares and sum-to-zero contrasts. When the interaction was not significant, the main effects of D and N were evaluated using an additive model with Type II sums of squares. When the interaction was significant, the main effects were not interpreted, and Holm-adjusted simple-effect comparisons were conducted.

To integrate relationships among morphological, physiological, and growth-related traits, a trait correlation network was constructed based on Spearman correlation coefficients and visualized using the corrplot package [50]. Partial least squares path modeling (PLS-PM), implemented with the plspm package, was further used to integrate environmental stress, oxidative damage, physiological defense, morphological change, and plant growth, thereby constructing a plant growth-defense trade-off pathway under multifactorial stress [51]. Other figures were primarily generated and refined using the ggplot2 package [52].

## 3. Results

### 3.1. Field Phenotypic Differentiation

Soil environmental factors across the 13 field sampling sites in the Songnen Plain showed pronounced spatial heterogeneity, forming a natural gradient from relatively low to high saline–alkaline intensity. Soil electrical conductivity (EC) ranged from 145.62 to 692.11 μS cm^−1^, and pH ranged from 8.62 to 10.08, indicating that the sampling sites covered alkaline to strongly alkaline habitats. Soil inorganic nitrogen content also varied markedly, with ${\mathrm{NH}}_{4}^{+}$-N ranging from 0.18 to 2.34 mg kg^−1^ and ${\mathrm{NO}}_{3}^{-}$-N ranging from 0.69 to 1.63 mg kg^−1^ (Table 1). Core environmental factors at each site were standardized using Z-scores and plotted in order of increasing soil EC (Figure 1B). The results showed that EC and pH, which represent habitat saline–alkaline intensity, increased highly synchronously across the spatial sequence, confirming the strong coupling between osmotic stress and a highly alkaline environment in soda saline–alkaline soils. However, the availability of inorganic nitrogen, including ${\mathrm{NH}}_{4}^{+}$-N and ${\mathrm{NO}}_{3}^{-}$-N, did not change linearly with the increasing saline–alkaline gradient but instead showed clear spatial fluctuations. This indicates spatial heterogeneity between saline–alkaline intensity and inorganic nitrogen availability in natural soda saline–alkaline wetlands.

Spearman correlation analysis (Figure 1C) preliminarily revealed the differential effects of abiotic environmental factors on aboveground–belowground phenotypic traits. EC was significantly positively correlated with population density (r=0.42, p<0.01), indicating that *B. planiculmis* can maintain relatively high population establishment at sites with higher EC. Soil ${\mathrm{NH}}_{4}^{+}$-N was significantly positively correlated with plant height (r=0.48, p<0.01) and basal diameter (r=0.58, p<0.001), suggesting a close association between ammonium nitrogen availability and individual plant growth status. In contrast, ${\mathrm{NO}}_{3}^{-}$-N was significantly negatively correlated with population density (r=−0.38, p<0.05). These patterns suggest that individual plant growth under saline–alkaline conditions is strongly constrained by the secondary effects of soil ammonium nitrogen availability.

To quantitatively evaluate the explanatory power of multiple environmental factors for overall community phenotypic variation, principal component analysis (PCA; Figure S1) and redundancy analysis (RDA; Figure 1D) were conducted. The RDA results showed that RDA1 and RDA2 explained 13.24% and 14.92% of the phenotypic variation, respectively, together accounting for 28.16% of the total variation. Therefore, the ordination should be interpreted as a partial explanation of field phenotypic differentiation rather than a complete representation of all environmental drivers. The alignment of EC and pH with population density, AGB, and BGB suggested that saline–alkaline intensity was associated with plant productivity and population establishment, whereas the position of ${\mathrm{NH}}_{4}^{+}$-N close to plant height and basal diameter supported its potential association with individual growth traits. Conversely, the vector of ${\mathrm{NO}}_{3}^{-}$-N was opposite to that of population density, which was consistent with the negative correlation between ${\mathrm{NO}}_{3}^{-}$-N and density observed in the Spearman correlation analysis.

### 3.2. Changes in Biomass Accumulation and Organ Resource Allocation

End-point measurements of substrate physicochemical properties confirmed that the greenhouse treatments generated distinct stress environments (Table S2). Compared with the CK, MS and SS markedly increased substrate pH and EC, with SS showing the highest pH and EC values. In contrast, PEG and XNPeg did not increase substrate EC or pH, but substantially decreased substrate water potential, indicating that these treatments mainly imposed osmotic limitation rather than saline–alkaline ion or high-pH stress. The XN showed substrate water potential values comparable to the CK, suggesting that this treatment primarily represented nitrogen deficiency without introducing additional osmotic or saline–alkaline stress. These results support the use of PEG and the XN as mechanistic reference treatments and confirm that soda saline–alkaline stress generated a more complex physicochemical environment involving elevated EC, increased pH, and reduced water potential.

**Figure 2:**
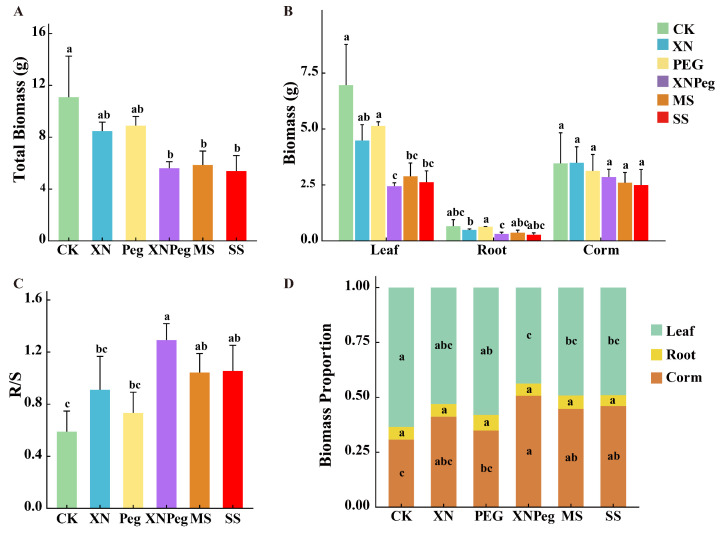
Effects of different treatments on biomass accumulation and allocation in *B. planiculmis*. (**A**) Total biomass under different treatments. (**B**) Biomass accumulation in different plant organs (leaves, roots, and corms). (**C**) R/S ratio in different treatments. (**D**) Biomass allocation proportions among organs. Treatments include the CK (control), XN (nitrogen deficiency), PEG (physiological drought), XNPeg (physiological drought and nitrogen deficiency), MS (moderate saline–alkaline stress), and SS (severe saline–alkaline stress). In panels (**A**–**C**), bars represent means, and upper error bars indicate one standard deviation (SD) above the mean. Different lowercase letters indicate significant differences among treatments within the same variable or organ (p<0.05).

The controlled greenhouse experiment showed that different treatments significantly altered biomass accumulation and organ allocation patterns in *B. planiculmis*, thereby modifying biomass allocation strategies (Figure 2). One-way ANOVA or Welch’s ANOVA showed that treatment had significant effects on total biomass, leaf biomass, root biomass, corm biomass, and R/S (p<0.05; Figure 2; Table S3). Compared with the CK, moderate saline–alkaline stress (MS) and severe saline–alkaline stress (SS) reduced total plant biomass by 47.18% and 51.32%, respectively (Table S3). In addition, the reductions in total biomass under MS and SS were clearly greater than those under physiological drought conditions alone (PEG) and were similar to those in the combined physiological drought and nitrogen deficiency treatment (XNPeg) (Figure 2A).

Biomass responses differed among organs. Within the saline–alkaline treatments, the proportion of leaf biomass decreased from approximately 63% for the CK to 49% under SS (Figure 2D). Although XNPeg showed an even lower leaf allocation proportion, it was interpreted as the combined physiological drought and nitrogen-deficiency reference treatment rather than as part of the saline–alkaline gradient (Figure 2D, Table S2). The value under SS was lower than that under MS, indicating that increasing saline–alkaline intensity further reduced aboveground biomass accumulation. Correspondingly, R/S increased from 0.59 for the CK to between 1.0 and 1.3 in saline–alkaline treatments. XNPeg also resulted in a higher R/S, whereas changes for PEG or the XN alone were relatively small (Figure 2C). Root biomass was lower than the CK for all stress treatments, with more pronounced decreases in saline–alkaline and XNPeg treatments. Corm biomass tended to decrease under saline–alkaline stress, especially under SS, although the differences among treatments were not significant in the post hoc comparison (Figure 2B). Therefore, saline–alkaline treatments suppressed the absolute biomass of both leaves and belowground organs, but the magnitude of reduction differed among organs. Organ allocation proportions further showed that saline–alkaline stress not only reduced absolute biomass, but also changed the relative allocation of biomass between aboveground and belowground organs.

### 3.3. Morphological Responses and Root Plasticity

**Figure 3:**
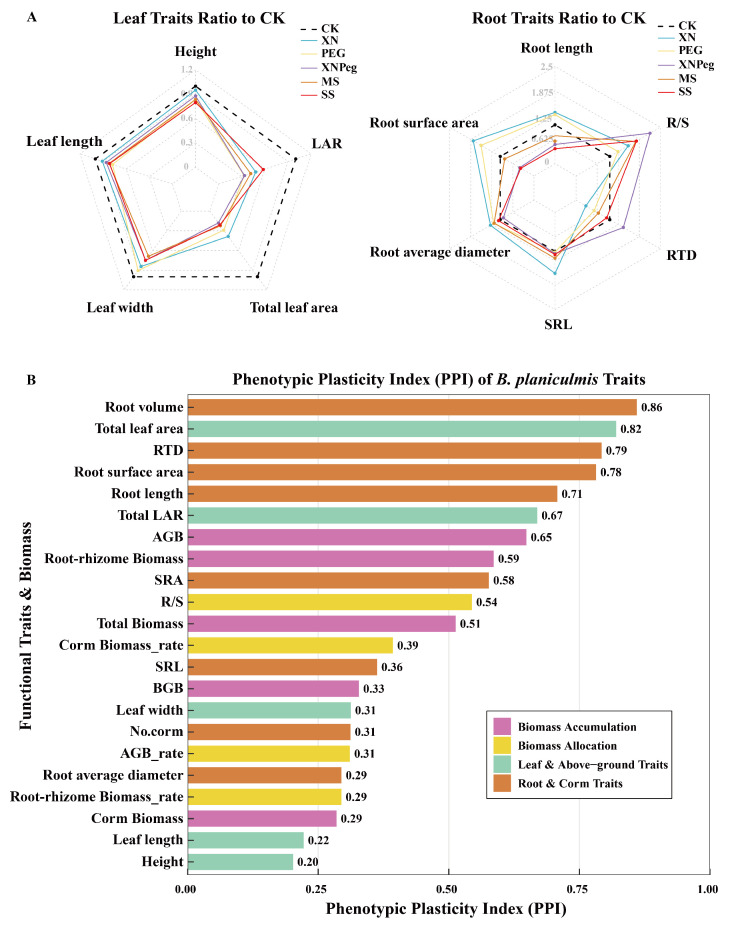
(**A**) Radar plots showing multidimensional changes in leaf and root morphological traits. (**B**) Ranking of phenotypic plasticity index (PPI) values for functional traits and biomass indicators.

Different treatments significantly altered aboveground morphology and root architecture in *B. planiculmis*. After standardization relative to the CK, radar plots showed that plant height, leaf length, leaf width, and total leaf area generally decreased in stress treatments. Saline–alkaline treatments, especially SS, exerted stronger inhibition on aboveground morphological traits. Compared with PEG or the XN alone, the relative values of multiple morphological traits were lower under MS and SS, indicating that saline–alkaline stress had a stronger effect on plant morphological development (Figure 3A).

Root traits showed high sensitivity to treatments. Under saline–alkaline stress, total root length, root surface area, and root volume were all lower than those for the CK, and the decrease was greater under SS, indicating that saline–alkaline stress mainly restricted spatial root expansion. In contrast to saline–alkaline stress, the single nitrogen deficiency treatment led to an increase in SRA, accompanied by an upward trend in SRL and a downward trend in RTD. However, MS and SS did not show a similar increase in SRA. Root expansion indicators were also significantly reduced for XNPeg, indicating that the root morphology was severely damaged after physiological drought combined with nitrogen deficiency, which is different from a single stress treatment (Figure 3A).

Analysis of the phenotypic plasticity index (PPI; Figure 3B) further showed that root volume, total leaf area, root tissue density (RTD), and root surface area exhibited the highest plasticity (PPI > 0.75), whereas basic structural traits such as plant height (PPI = 0.20) and leaf length (PPI = 0.22) were relatively stable. This indicates that *B. planiculmis* mainly adapts to saline–alkaline and other abiotic stress environments through highly dynamic adjustments in root morphology.

### 3.4. Interactions Between Physiological Drought and Nitrogen Deficiency and Comparison with Saline–Alkaline Physiological Responses

**Figure 4:**
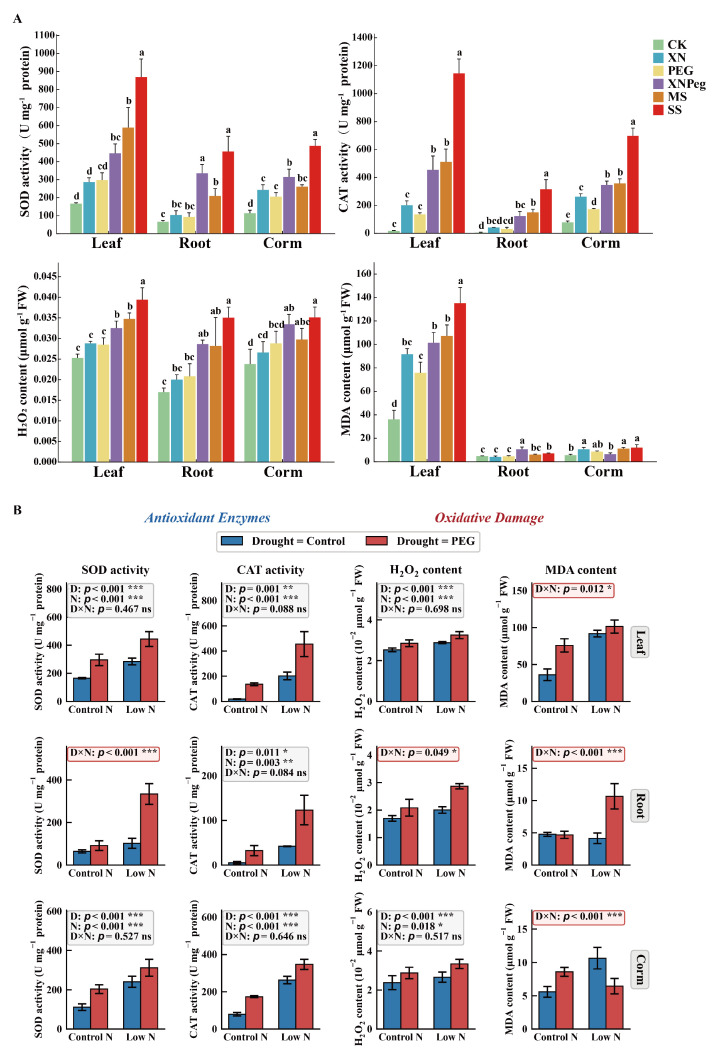
Physiological responses of *B. planiculmis* to different stresses and interactions between physiological drought and nitrogen deficiency. (**A**) Accumulation levels of SOD, CAT, H_2_O_2_, and MDA in all treatment groups (CK, PEG, XN, XNPeg, MS, and SS). Bars represent means, and upper error bars indicate one standard deviation (SD) above the mean. Different lowercase letters indicate significant differences among treatments (p<0.05). (**B**) Grouped bar plots showing the effects of physiological drought and nitrogen deficiency under the CK, PEG, XN, and XNPeg treatments. Bars represent means ± SD based on the available valid biological replicates. D, N, and D × N represent the effects of physiological drought, nitrogen deficiency, and their interaction, respectively. When the D × N interaction was significant, only the interaction effect is presented and simple effects were subsequently examined. When the interaction was not significant, the main effects of D and N are presented. Significance levels for main effects and interactions are indicated as follows: * p<0.05, ** p<0.01, *** p<0.001, and ns, not significant.

Different treatments altered antioxidant enzyme activities and oxidative damage levels in the leaves, roots, and corms of *B. planiculmis* (Figure 4A; Table S4). Compared with the CK, nitrogen deficiency alone (XN) and PEG-induced physiological drought generally increased SOD and CAT activities and elevated $H_{2}$$O_{2}$ and MDA contents, although the magnitude and statistical significance of these responses varied among organs and indicators. These results indicate that both nitrogen deficiency and osmotic limitation can induce oxidative pressure and activate antioxidant defense. However, the responses to PEG or XN alone were generally weaker than those observed under the combined XNPeg treatment and the saline–alkaline treatments, indicating that neither physiological drought nor nitrogen deficiency alone fully explained the physiological injury caused by soda saline–alkaline stress.

Two-way ANOVA revealed significant interactions between physiological drought and nitrogen deficiency for leaf MDA (p=0.012), root SOD (p<0.001), root $H_{2}$$O_{2}$ (p=0.049), root MDA (p<0.001), and corm MDA (p<0.001), whereas the interactions were not significant for the remaining variables (Figure 4B). Holm-adjusted simple-effect analyses showed that PEG increased leaf MDA under control-nitrogen conditions but not under nitrogen-deficiency conditions. In roots, PEG significantly increased SOD activity, $H_{2}$$O_{2}$ content, and MDA content under nitrogen-deficiency conditions, whereas nitrogen deficiency significantly increased these variables under PEG treatment. These results indicate that the combination of osmotic limitation and nitrogen deficiency amplified antioxidant activation and oxidative damage in roots. Corm MDA showed a crossover interaction, with PEG increasing MDA under control-nitrogen conditions but decreasing it under nitrogen-deficiency conditions. For variables without a significant interaction, physiological drought and nitrogen deficiency both had significant positive main effects on leaf SOD activity, leaf CAT activity, leaf $H_{2}$$O_{2}$ content, root CAT activity, corm SOD activity, corm CAT activity, and corm $H_{2}$$O_{2}$ content.

Under severe saline–alkaline stress (SS), higher antioxidant enzyme activities and oxidative damage levels were observed in leaves and roots, indicating that increasing saline–alkaline intensity aggravated reactive oxygen species accumulation and membrane lipid peroxidation. The simultaneous increases in SOD and CAT activities and in $H_{2}$$O_{2}$ and MDA contents indicate that antioxidant defense was activated but was insufficient to fully offset oxidative damage. Compared with the mechanistic decoupling treatments, MS and SS generally produced stronger responses than nitrogen deficiency or PEG alone and were similar to XNPeg for some indicators. However, the physiological responses under saline–alkaline stress were not identical to those under XNPeg, indicating that soda saline–alkaline injury was associated not only with osmotic limitation and nitrogen deficiency, but also with high pH and saline–alkaline ion toxicity.

### 3.5. Exogenous Nitrogen Acquisition and Organ Allocation

**Figure 5:**
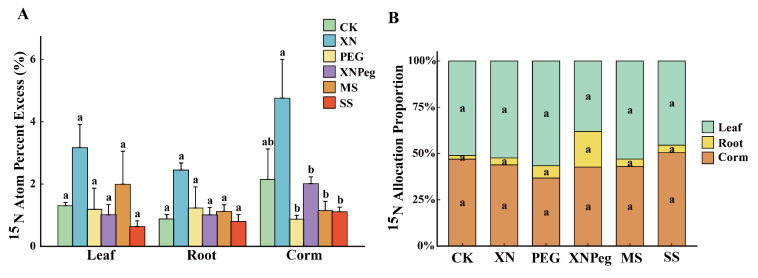
Nitrogen acquisition and allocation strategies of *B. planiculmis* for different stress treatments based on ^15^N isotope tracing. (**A**) Atom percent excess of ^15^N (%) in leaves, roots, and corms, reflecting nitrogen enrichment in plant tissues. Bars represent means, and upper error bars indicate one standard error (SE) above the mean. (**B**) Allocation proportions of absorbed ^15^N among different organs. Treatments include the CK, PEG, XN, XNPeg, MS, and SS. Different lowercase letters indicate significant differences among treatments within the same organ (p<0.05).

The ^15^N tracing results showed that different treatments significantly altered the acquisition of exogenous nitrogen by *B. planiculmis* and its allocation among organs (Figure 5, Table S5). Under nitrogen deficiency conditions alone (XN), ^15^N APE was generally higher in all organs, with the highest APE observed in corms. This indicates that nitrogen deficiency can induce plants to enhance the enrichment of exogenous labeled nitrogen and promote the entry of the labeled nitrogen signal into belowground storage organs. In contrast, the ^15^N APE in leaves, roots, and corms was generally lower under moderate saline–alkaline stress (MS) and severe saline–alkaline stress (SS), with a significant decrease in corm APE in saline–alkaline treatments. These results indicate that saline–alkaline stress did not induce a compensatory nitrogen uptake response similar to that under nitrogen deficiency conditions alone but instead restricted the entry of exogenous labeled nitrogen into the plant and its enrichment in corms. This inhibition may be jointly related to restricted root expansion, osmotic limitation, high pH, and ion injury under saline–alkaline stress.

The ^15^N allocation proportions showed that, across treatments, absorbed labeled nitrogen was mainly allocated to leaves and corms, while the root fraction was relatively low (Figure 5B). Under saline–alkaline stress, corms still maintained a relatively high proportion of ^15^N allocation, particularly under SS, where corms accounted for approximately half of the labeled nitrogen. This suggests that *B. planiculmis* retains a tendency to allocate limited nitrogen to belowground storage organs under saline–alkaline stress. It should be noted that a high relative allocation proportion to corms does not indicate an increase in absolute nitrogen accumulation in corms. Considering the reductions in corm biomass and corm APE under saline–alkaline stress, saline–alkaline stress may overall weaken nitrogen input into underground corm resources.

### 3.6. PLS-PM Integration Suggests Potential Growth-Limitation Pathways Under Stress

**Figure 6:**
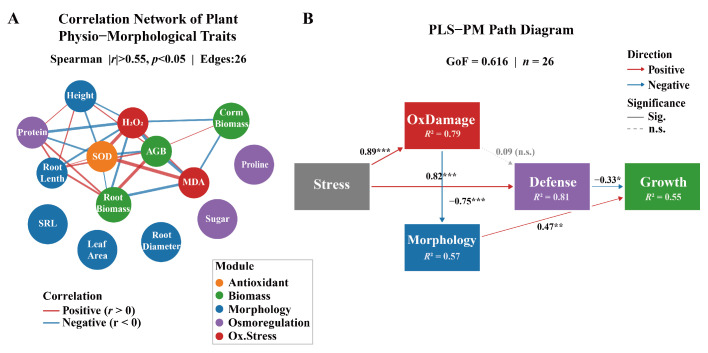
System-level regulation and physiological trade-off mechanisms of plant growth under stress conditions. (**A**) Spearman correlation network of plant physiological and morphological traits. Node colors represent different functional modules (antioxidant defense, biomass, morphology, osmotic adjustment, and oxidative stress), and lines indicate significant correlations (|r|>0.55, p<0.05; red lines indicate positive correlations, and blue lines indicate negative correlations). (**B**) Partial least squares path model (PLS-PM) showing potential causal relationships among environmental stress, oxidative damage (OxDamage), physiological defense (Defense), morphological adjustment (Morphology), and overall plant growth (Growth). Red arrows indicate positive effects, blue arrows indicate negative effects, and dashed lines indicate non-significant paths. Path coefficients are shown beside the arrows (* p<0.05, ** p<0.01, *** p<0.001, and n.s., not significant). GoF, goodness of fit; $R^{2}$, coefficient of determination, indicating the proportion of variance explained by the model.

To further integrate the relationships among environmental stress, oxidative damage, physiological defense, morphological adjustment, and plant growth, we constructed a Spearman correlation network and a partial least squares path model (PLS-PM) (Figure 6). The correlation network showed relatively clear modular associations among functional traits. Oxidative damage indices, such as MDA and $H_{2}$$O_{2}$, were mainly negatively correlated with biomass-related indicators and some root morphological traits, whereas antioxidant enzyme indicators, such as SOD and CAT, were closely associated with oxidative damage indices (Figure 6A).

PLS-PM path analysis (GoF = 0.616) suggested that the relationships among environmental stress, oxidative damage, defense responses, morphology, and growth were consistent with two potential growth-limitation pathways. First, stress directly induced strong oxidative damage (OxDamage; path coefficient β=0.887), which then indirectly suppressed biomass accumulation by reducing root morphological development (β=−0.753). Second, stress activated defense responses (β=0.819), but biomass remained significantly inhibited in a high-intensity defense state (β=−0.332). The morphological adaptation latent variable, represented by root length and plant height, was the only factor with a positive direct effect on growth (β=0.470). This suggests that maintaining root morphological integrity under saline–alkaline stress has a more direct influence on biomass allocation than simply increasing osmotic adjustment compound concentrations.

## 4. Discussion

### 4.1. Phenotypic Differentiation and Belowground Resource Allocation Under Soda Saline–Alkaline Environmental Filtering Conditions

The field survey showed that natural *B. planiculmis* distribution areas in the Songnen Plain exhibited a distinct soda saline–alkaline environmental gradient. EC and pH generally increased synchronously across the site gradient, whereas ${\mathrm{NH}}_{4}^{+}$-N and ${\mathrm{NO}}_{3}^{-}$-N did not show a simple linear pattern with saline–alkaline intensity. This indicates that natural soda saline–alkaline wetlands are not defined by a single salinity gradient, but instead represent heterogeneous habitats jointly shaped by salinity, alkalinity, hydrological status, and nutrient availability. Previous studies have shown that soda saline–alkaline stress usually involves osmotic limitation, ${\mathrm{Na}}^{+}$/${\mathrm{HCO}}_{3}^{-}$/${\mathrm{CO}}_{3}^{2-}$ ion toxicity, high-pH stress, and reduced nutrient availability; therefore, its effects on plants are often more complex than those of neutral salt stress [3,53].

The positive relationship between *B. planiculmis* density and EC indicates that this species retains the capacity to establish and maintain populations under relatively high saline–alkaline conditions. This supports previous evidence that *B. planiculmis* is a facultative salt-tolerant wetland species capable of persisting in the soda saline–alkaline wetlands of the Songnen Plain [6,11,54,55]. However, the first two axes of RDA only explained 28.16% of the total phenotypic variation, indicating that the measured variables only captured a portion of the field scale heterogeneity. In natural soda saline alkali wetlands, unmeasured hydrological variables, such as water depth, soil moisture, and flooding duration, may further regulate water-salt redistribution and nutrient availability [8,56]. Therefore, the RDA results should be interpreted as identifying associations between measured soil properties and plant traits rather than fully explaining all sources of phenotypic variation in natural wetlands. In addition, the contrasting relationships of ${\mathrm{NH}}_{4}^{+}$-N and ${\mathrm{NO}}_{3}^{-}$-N with plant traits suggest that inorganic nitrogen forms may have different ecological implications for this species. ${\mathrm{NH}}_{4}^{+}$-N was associated with individual growth traits, whereas ${\mathrm{NO}}_{3}^{-}$-N was negatively related to population density. In wetland environments, nitrate dynamics are strongly affected by water level, redox conditions, and saline–alkaline microenvironments, and elevated ${\mathrm{NO}}_{3}^{-}$-N may not always indicate more favorable nitrogen supply [11,57,58,59]. Therefore, *B. planiculmis* in saline–alkaline wetlands may exhibit a strategy of population maintenance under constrained individual performance conditions.

The controlled greenhouse experiment should be regarded as a mechanistic decoupling of major stress components rather than a complete simulation of field conditions. It further showed that saline–alkaline stress caused stronger growth inhibition than either physiological drought or nitrogen deficiency alone. This indicates that growth suppression under soda saline–alkaline stress cannot be attributed solely to osmotic limitation or nitrogen shortage. Instead, it reflects a compound limitation involving water uptake restriction, impaired nutrient acquisition, high pH, and ion toxicity. The increase in root-to-shoot ratio under saline–alkaline stress suggests a relative shift toward belowground allocation, a common response when plants experience resource or water limitation [60,61,62]. However, because absolute root and corm biomass also decreased, this shift should be interpreted as relative maintenance of belowground organs under overall growth suppression, rather than as an actual expansion of the belowground resource pool.

Root morphological results further explain this change in resource allocation. Under nitrogen deficiency conditions alone, SRA increased, accompanied by tendencies toward increased SRL and decreased RTD. This indicates that, under nutrient deficiency conditions alone, *B. planiculmis* tends to form thinner and longer roots with a larger absorptive area per unit biomass to enhance nitrogen acquisition. This pattern is consistent with the low-construction-cost and high-resource-acquisition strategy described in the root economics space framework [14,16,18,63,64,65]. In contrast, saline–alkaline stress reduced root length, surface area, and volume without inducing a similar increase in SRA. Thus, saline–alkaline stress did not promote a typical nutrient-foraging response but instead constrained root spatial development. This distinction is important because it indicates that the limiting effect of saline–alkaline stress on *B. planiculmis* operates not only through reduced nutrient availability but also through direct inhibition of root formation and expansion. Consequently, the species may retain some belowground regenerative capacity under stress, but its ability to acquire resources and build corm reserves is substantially restricted.

### 4.2. Physiological Damage and Nitrogen Resource Acquisition Under Saline–Alkaline Stress

Physiological measurements and ^15^N tracing revealed that saline–alkaline stress constrained *B. planiculmis* growth through coupled oxidative damage and impaired nitrogen acquisition. In this study, the increase in SOD and CAT activities indicates activation of antioxidant defense, whereas the simultaneous accumulation of $H_{2}$$O_{2}$ and MDA shows that reactive oxygen species production and membrane lipid peroxidation exceeded the plant’s buffering capacity [3,66,67,68]. Therefore, *B. planiculmis* under saline–alkaline stress was in a state of activated defense accompanied by persistent oxidative injury.

The mechanistic decoupling treatments helped distinguish the contributions of physiological drought and nitrogen deficiency to this injury. Both factors induced oxidative pressure, but their combined effects varied among organs and indicators rather than being uniformly additive. Their combined effect was most evident in roots, where antioxidant activation and oxidative damage were enhanced, whereas leaf and corm MDA showed distinct response patterns. This is biologically plausible because nitrogen is required for protein synthesis, enzyme maintenance, photosynthetic metabolism, and tissue repair, while physiological drought restricts water uptake and promotes ROS accumulation [4,12,13,69,70]. Nevertheless, soda saline–alkaline stress was not equivalent to the simple combination of PEG-induced physiological drought and nitrogen deficiency. The presence of high pH, ${\mathrm{Na}}^{+}$, ${\mathrm{HCO}}_{3}^{-}$, and ${\mathrm{CO}}_{3}^{2-}$ may further disrupt pH homeostasis, ion balance, membrane stability, and transmembrane transport [2,3,4,71]. This may explain why several physiological responses under MS and SS differed from those for XNPeg and supports the view that soda saline–alkaline stress represents a distinct compound stress rather than a direct sum of osmotic limitation and nutrient deficiency.

The ^15^N tracing results further showed that saline–alkaline stress weakened exogenous nitrogen enrichment and reduced nitrogen input into corms. Under nitrogen deficiency conditions alone, higher ^15^N enrichment was consistent with the acquisitive root adjustment described above, suggesting a compensatory nitrogen uptake response. In contrast, saline–alkaline treatments did not induce such compensation but instead limited the entry of labeled nitrogen into plants and its subsequent enrichment in corms [58,72]. This limitation likely resulted from the combined effects of restricted root expansion, osmotic stress, high-pH interference, and reduced nitrogen-use efficiency in soda saline–alkaline conditions [4,13,71]. Importantly, corms still maintained a relatively high proportion of absorbed ^15^N allocation under saline–alkaline stress. This suggests that *B. planiculmis* tends to preserve some nitrogen allocation to belowground storage organs when external nitrogen acquisition is constrained. Similar organ-level trade-offs in carbon and nitrogen allocation have been reported for *B. planiculmis* under saline–alkaline conditions [11], and nitrogen enrichment or competition can also alter belowground investment in this species [59]. More broadly, corms and other belowground storage organs function as reserve structures that support regrowth, clonal persistence, and survival under unfavorable conditions [73]. For corm-producing or storage-organ-forming plants, salt or alkaline stress may therefore create a trade-off between stress defense and reserve renewal: resources are required for osmotic adjustment and antioxidant protection, while the formation or maintenance of belowground reserves may be constrained [74,75]. In this context, the relatively high corm allocation proportion observed in *B. planiculmis* may reflect a conservative reserve-maintenance strategy. However, the simultaneous decline in corm ^15^N enrichment and absolute corm biomass indicates that relative allocation priority is insufficient to maintain the absolute corm resource pool. Thus, the physiological response of *B. planiculmis* to saline–alkaline stress is best understood as a defense-activation and resource-reallocation process under compound stress, rather than as a simple drought-adaptation or nitrogen-deficiency response.

### 4.3. Implications of Growth–Defense Trade-Offs for the Maintenance of Waterbird Food Resources

The correlation network and PLS-PM analysis integrated the morphological, physiological, and nitrogen-related responses into a common framework. However, because ionic toxicity, ionic homeostasis, and other unmeasured stress components were not directly included, and because the number of biological replicates was relatively limited, the PLS-PM results should be interpreted as an exploratory summary of coordinated trait relationships rather than definitive causal estimates. The model suggested that environmental stress was positively associated with oxidative damage and defense responses, while oxidative damage constrained morphological development and consequently reduced growth. Defense responses were also negatively associated with growth, suggesting a potential cost of stress resistance. Together, these results indicate that the decline in *B. planiculmis* growth under saline–alkaline stress was not driven by a single trait but by a coordinated process involving oxidative injury, defense costs, root limitation, and reduced nitrogen acquisition.

This pattern is consistent with the theory of growth–defense trade-offs, which proposes that enhanced defense under resource limitation conditions can reduce the resources available for growth and structural development [20,21,22,76,77,78]. In the present study, increased antioxidant enzyme activities, persistent oxidative damage, restricted root expansion, reduced ^15^N enrichment, and lower biomass together indicate a saline–alkaline-induced growth–defense trade-off. In this strategy, *B. planiculmis* maintained some relative allocation to belowground organs but at the cost of reduced overall growth and weakened corm resource formation. Therefore, belowground maintenance in this species should be viewed as a relative allocation tendency under stress, not as evidence that corm production remains stable.

This distinction has important implications for waterbird habitat assessment. The corms of *B. planiculmis* are both storage and vegetative reproductive organs of the plant and important belowground food resources for Siberian Cranes and other migratory waterbirds during stopover periods [7,9,79,80]. Wetland restoration and habitat evaluation often rely on vegetation-based indicators, such as target plant occurrence, community cover, or aboveground biomass. However, such indicators may not accurately reflect the availability or quality of belowground food resources, especially in sedge-dominated wetlands where aboveground and belowground production can respond differently to environmental stress [81,82]. The present findings show that *B. planiculmis* may persist as a population and retain a relatively high corm allocation proportion even when absolute corm biomass and nitrogen input decline. From a plant-level perspective, our results suggest that plant occurrence or aboveground vegetation recovery alone may not fully reflect stress-induced changes in belowground corm resources. However, because bird responses, field-scale corm availability, and corm nutritional quality were not directly assessed in this study, these habitat-related implications should be regarded as hypotheses and considerations for future assessments.

These plant-level implications should be interpreted with several constraints in mind. The field survey did not include key hydrological variables, such as water depth, soil moisture, and flooding duration, which may regulate water–salt redistribution and nutrient availability in natural wetlands. The greenhouse experiment represented a short-term early-stage response rather than a complete seasonal or perennial growth cycle; although clonal ramets and corm expansion were already observed by harvest, longer-term studies are needed to evaluate mature corm storage and perennial population maintenance. In addition, phosphorus dynamics, plant ionomic or micronutrient composition, direct plant water status, and independent rhizome development were not fully assessed. Because field-scale corm availability, corm nutritional quality, and waterbird responses were also not directly measured, future studies should combine longer-term field experiments with plant nutrient and ionomic analyses, rhizome–corm monitoring, and habitat-use observations to better link plant stress physiology with wetland habitat function.

## 5. Conclusions

This study showed that soda saline–alkaline stress strongly constrained the growth of *B. planiculmis* by restricting root expansion, increasing oxidative damage and defense costs, and reducing nitrogen acquisition and allocation to belowground organs. Comparisons with PEG-induced physiological drought, nitrogen deficiency, and their combined treatment further indicated that soda saline–alkaline stress is not a simple sum of osmotic limitation and nitrogen deficiency but a distinct compound stress involving multiple interacting constraints. Although *B. planiculmis* maintained a relatively high allocation proportion to belowground corms under stress, absolute corm biomass and nitrogen input declined, suggesting that relative allocation patterns do not necessarily indicate stable belowground food-resource formation. These findings provide plant-level evidence that stress-induced changes in corm resources should be considered when evaluating saline–alkaline wetland condition. Future studies should combine longer-term field experiments with measurements of plant nutrient status, rhizome and corm development, plant water relations, and waterbird use to better link plant stress physiology with wetland habitat function.

## Figures and Tables

**Table 1:** Physicochemical properties and plant phenotypes of the field sampling sites.

No.	Location	Environment				Plants					
		EC (μS/cm)	pH	${\mathrm{NH}}_{4}^{+}$-N (mg/kg)	${\mathrm{NO}}_{3}^{-}$-N (mg/kg)	Density (plants/m^2^)	Height (cm)	Diameter (cm)	AGB (g/m^2^)	BGB (g/m^2^)	R/S
1	BHH	232.2 ± 1.0 ij	9.01 ± 0.01 i	0.49 ± 0.14 bcd	0.92 ± 0.06 c	362.7 ± 113.5 ab	67.9 ± 7.8 ab	0.31 ± 0.05 d	308.6 ± 81.4 bc	349.0 ± 31.8 ab	1.21 ± 0.47
2	BXZ	602.3 ± 12.0 d	9.61 ± 0.02 d	0.83 ± 0.12 bc	1.03 ± 0.07 bc	576.0 ± 184.5 ab	70.4 ± 15.2 ab	0.70 ± 0.13 a	705.5 ± 200.4 a	345.2 ± 188.7 ab	0.54 ± 0.32
3	CHM	145.6 ± 0.7 l	8.62 ± 0.02 k	0.40 ± 0.13 bcd	1.50 ± 0.18 ab	112.0 ± 57.7 b	27.1 ± 2.1 c	0.32 ± 0.15 d	101.9 ± 8.5 c	127.6 ± 32.5 b	1.26 ± 0.33
4	CL	218.1 ± 1.0 j	8.95 ± 0.04 j	0.87 ± 0.37 bc	1.05 ± 0.28 bc	298.7 ± 51.4 ab	49.8 ± 8.4 abc	0.64 ± 0.10 ab	216.8 ± 69.4 bc	192.6 ± 54.0 b	1.03 ± 0.65
5	HBT3	233.3 ± 1.2 i	8.91 ± 0.03 j	0.92 ± 0.18 b	0.85 ± 0.03 c	720.0 ± 32.0 a	77.3 ± 23.2 a	0.51 ± 0.14 abcd	688.6 ± 87.1 a	914.3 ± 590.4 a	1.35 ± 0.87
6	HBT4	532.8 ± 6.5 e	9.92 ± 0.01 b	0.77 ± 0.17 bcd	0.95 ± 0.09 c	458.7 ± 51.4 ab	74.9 ± 12.0 a	0.62 ± 0.12 abc	214.1 ± 66.6 bc	189.4 ± 97.4 b	0.84 ± 0.20
7	QTP	458.1 ± 4.2 f	9.37 ± 0.01 f	2.34 ± 0.24 a	1.63 ± 0.07 a	458.7 ± 104.1 ab	54.2 ± 16.4 abc	0.51 ± 0.08 abcd	243.1 ± 105.5 bc	335.0 ± 42.2 ab	1.57 ± 0.67
8	SL	671.1 ± 6.6 b	9.91 ± 0.01 b	0.18 ± 0.06 d	1.63 ± 0.45 a	309.3 ± 106.5 ab	38.7 ± 17.6 abc	0.34 ± 0.04 cd	225.6 ± 112.3 bc	307.5 ± 166.8 ab	1.34 ± 0.43
9	SMC1	404.4 ± 2.7 g	9.52 ± 0.02 e	0.35 ± 0.29 bcd	0.69 ± 0.02 c	773.3 ± 175.5 a	47.2 ± 7.4 abc	0.26 ± 0.06 d	303.9 ± 125.2 bc	744.3 ± 145.4 ab	2.60 ± 0.67
10	SMC2	692.1 ± 3.2 a	10.08 ± 0.01 a	0.38 ± 0.24 bcd	0.86 ± 0.24 c	688.0 ± 277.6 a	33.3 ± 4.5 bc	0.39 ± 0.09 bcd	263.4 ± 111.4 bc	377.9 ± 222.5 ab	1.67 ± 1.08
11	SMC3	307.8 ± 4.5 h	9.14 ± 0.01 h	0.34 ± 0.09 bcd	0.79 ± 0.08 c	570.7 ± 128.3 ab	32.0 ± 6.5 bc	0.36 ± 0.08 bcd	196.5 ± 14.8 bc	279.8 ± 7.0 ab	1.43 ± 0.09
12	TL	182.7 ± 0.6 k	9.22 ± 0.02 g	0.32 ± 0.16 cd	0.78 ± 0.02 c	293.3 ± 37.0 ab	46.0 ± 19.0 abc	0.35 ± 0.06 bcd	220.0 ± 50.0 bc	194.2 ± 44.0 b	0.95 ± 0.45
13	WLZ	639.4 ± 4.5 c	9.71 ± 0.01 c	0.87 ± 0.20 bc	0.86 ± 0.07 c	680.0 ± 472.0 a	40.2 ± 12.9 abc	0.35 ± 0.10 bcd	493.2 ± 254.4 ab	425.2 ± 497.4 ab	0.91 ± 1.09

Note: Values are means ± SD (n=3). Different lowercase letters within the same column indicate significant differences among sampling sites (p<0.05). Variables without lowercase letters showed no significant differences among sampling sites. For each variable, homogeneity of variance was first tested using Levene’s test. One-way ANOVA followed by Tukey’s HSD test was used when variances were homogeneous; Welch’s ANOVA followed by Games–Howell test was used when variances were heterogeneous. Sampling site abbreviations are as follows: BHH, Baihe Lake; BXZ, Baxizhao; CHM, Caihongmen; CL, Caili; HBT3, Hubutai site 3; HBT4, Hubutai site 4; QTP, Qiantaiping; SL, Shaoli; SMC1, Sanmachang site 1; SMC2, Sanmachang site 2; SMC3, Sanmachang site 3; TL, Taolagao; WLZ, Wulanzhao. AGB, aboveground biomass; BGB, belowground biomass; R/S, root-to-shoot ratio, calculated using BGB/AGB.

## Data Availability

Data are contained within the article and Supplementary Materials.

## Supplementary Materials

The following supporting information can be downloaded at https://www.mdpi.com/article/10.3390/biology15151255/s1: Table S1. Nutrient ion content of Hoagland complete nutrient solution and nutrient deficient nutrient solution (μmol); Table S2. Substrate physicochemical properties at harvest under different greenhouse treatments; Table S3. Effects of different treatments on *B. planiculmis* morphological and biomass traits; Table S4. Effects of different treatments on physiological and biochemical indicators in various organs of *B. planiculmis*; Table S5. Effects of different treatments on ^15^N absolute abundance and allocation proportion in various organs of *B. planiculmis*.
